# Training Trainers in health and human rights: Implementing curriculum change in South African health sciences institutions

**DOI:** 10.1186/1472-6920-11-47

**Published:** 2011-07-25

**Authors:** Elena G Ewert, Laurel Baldwin-Ragaven, Leslie London

**Affiliations:** 1 Denver Health Residency in Emergency Medicine, MC #0108 777 Bannock St, Denver, Colorado 80206, USA; 2 Asylum Hill Family Practice Center, 99 Woodland Street, Hartford, Connecticut 06105, USA; 3 School of Public Health and Family Medicine, Falmouth Building, Faculty of Health Sciences, University of Cape Town, Observatory, 7925, South Africa

## Abstract

**Background:**

The complicity of the South African health sector in apartheid and the international relevance of human rights as a professional obligation prompted moves to include human rights competencies in the curricula of health professionals in South Africa. A Train-the-Trainers course in Health and Human Rights was established in 1998 to equip faculty members from health sciences institutions nationwide with the necessary skills, attitudes and knowledge to teach human rights to their students. This study followed up participants to determine the extent of curriculum implementation, support needed as well as barriers encountered in integrating human rights into health sciences teaching and learning.

**Methods:**

A survey including both quantitative and qualitative components was distributed in 2007 to past course participants from 1998-2006 via telephone, fax and electronic communication.

**Results:**

Out of 162 past participants, 46 (28%) completed the survey, the majority of whom were still employed in academic settings (67%). Twenty-two respondents (48%) implemented a total of 33 formal human rights courses into the curricula at their institutions. Respondents were nine times more likely (relative risk 9.26; 95% CI 5.14-16.66) to implement human rights education after completing the training. Seventy-two extracurricular activities were offered by 21 respondents, many of whom had successfully implemented formal curricula. Enabling factors for implementation included: prior teaching experience in human rights, general institutional support and the presence of allies - most commonly coworkers as well as deans. Frequently cited barriers to implementation included: budget restrictions, time constraints and perceived apathy of colleagues or students. Overall, respondents noted personal enrichment and optimism in teaching human rights.

**Conclusion:**

This Train-the-Trainer course provides the historical context, educational tools, and collective motivation to incorporate human rights educational initiatives at health sciences institutions. Increased implementation of human rights instruction, both formally and extracurricularly, has demonstrated the training's significance not only within academic institutions but more broadly across the health sector. Coworkers are vital allies in teaching human rights to health sciences students, helping to alleviate institutional barriers. Training fellow staff members and those in key leadership roles is noted as vital to the sustainability of human rights education.

## Background

Any discussion of health and human rights in South Africa must acknowledge that country's history and legacy of apartheid. Prolonged colonial occupation by the Dutch and English gave way to various forms of legalized segregation, whereby the White minority secured complete power and control of the country in 1948 [1]. In addition to the denial of voting rights for Black, Coloured (those of mixed 'racial' descent) and Indian people, restricted educational opportunities for 'non-Europeans', physical relocation of millions on the basis of 'racial' categorization through the notorious Group Areas Act (See Additional File 1), and racially segregated amenities (such as separate bathrooms for different 'race' groups), the apartheid government applied discriminatory policies throughout the healthcare system. In fact, it has been said that, "if an outsider wished to know what 'apartheid' was, an examination of health and health care would have given them excellent insight into this system of segregation, inequality and oppression." [2] Many health care professionals participated in and perpetuated apartheid through both active means and passive acceptance [3-6]. Throughout South African society, the systematic distribution of land and wealth along 'racial' lines and lack of access to resources for the majority of the population continued to increase. In 1994, the year suffrage was extended to everyone, South Africa had one of the highest per capita mortality rates in the world, resulting in part from extremely high rates of health and economic inequalities, rising burdens of communicable and non-communicable disease, as well as the traumatic consequences of widespread violence [7].

In its move to democracy, South Africa adopted a transitional justice approach to what the United Nations General Assembly termed "a crime against humanity" when referring to the years of racial injustice and appalling human rights violations that occurred under apartheid [8]. The Truth and Reconciliation Commission (TRC), which held its first public hearings in May 1996, sought to understand the full spectrum of gross human rights violations under apartheid. It did this through three mechanisms: collecting statements of violations, granting amnesty to perpetrators in exchange for full disclosure, and recommending reparations and rehabilitation for victims. During the Special Hearings on the role of the health sector under apartheid, the TRC found that:

"the health sector, through apathy, acceptance of the status quo and acts of omission, allowed the creation of an environment in which the health of millions of South Africans was neglected, even at times actively compromised, and in which violations of moral and ethical codes of practice were frequent, facilitating violations in human rights." [9]

In addition to highlighting human rights abuses committed by the health professions, the TRC outlined the role of statutory councils, such as the South African Nursing Council (SANC) and the South African Medical and Dental Council (SAMDC), and other health professional organisations in failing to hold their members accountable for their actions. Realising that a culture of impunity begins with the earliest training, the TRC called for the promotion and integration of human rights into the curricula of all medical and allied health schools. The Commission furthermore recommended that a health and human rights body be established, which, among other things, would advise on educational matters in health and human rights [10-13].

In an effort to address the challenges of implementing a human rights approach across health professional training in South Africa, the University of Cape Town (UCT) and the Cape Town-based Trauma Centre for Survivors of Violence and Torture spearheaded a two-year national initiative (1997 - 1999), called the Health and Human Rights Project (HHRP). The HHRP provided research, support and input for the TRC's Special Hearings on the Health Sector. In promoting human rights in the health sector, the HHRP also led to the formation of the Health and Human Rights Programme in the School of Public Health and Family Medicine at UCT [14]. To promote the adoption of health and human rights training and advocacy by other higher education institutions, a Train-the-Trainers course on Health and Human Rights for Health Professional Educators was established in 1998 [15,16].

**Figure 1:**
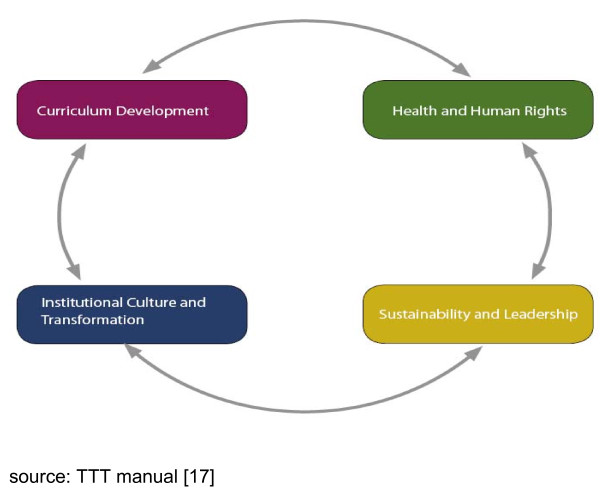
**Train-the-Trainers thematic integration**.

The Train the Trainers (TTT) course has been held annually from 1998, with a brief hiatus in 1999. Taught as a short course over five consecutive weekdays (typically during university holidays), the TTT course aims to foster proficiency in health and human rights for instructors in the health sector [17]. Learning objectives are arranged around four inter-related themes, which include: the context and content of health and human rights, foundations of curriculum development, issues in institutional transformation, and leadership and sustainability in teaching and learning health and human rights (Figure 1). Table 1 further lists the learning objectives of the TTT course by theme.

**Table 1:** Learning objectives of the Train the Trainers course by theme^17^

1	*Health and Human Rights *seeks to assist health professionals to:
•	Understand the conceptual framework for human rights, and its relationship to health
•	Understand the historical context as well as national and international human rights debates relating health to human rights
•	Promote an understanding of professional and ethical codes to support human rights

2	*Institutional Transformation *seeks to assist health professionals to:
•	Understand the role of the health sector under apartheid
•	Explore past, current, and future roles of the health sector in respect of human rights
•	Recognize the importance of self-study and reflection

3	*Curriculum Development for Human Rights *seeks to assist health professionals to:
•	Explore strategies for curriculum change, including multidisciplinary teaching and identifying clinical settings in which human rights abuses take place
•	Explore the relationship between ethics and human rights
•	Identify core and discipline-specific competencies
•	Share available resources for teaching (electronic and other)
•	Develop training materials

4	*Sustainability and Leadership *seeks to assist health professionals to:
•	Foster a supportive network to continue to integrate education and training in health and human rights
•	Engage with processes that set educational standards for the training of health professionals and national and international human rights debates relating health and human rights.

Different teaching methods are employed throughout the course. In didactic sessions, participants learn about international human rights law and the South African context for why training healthcare professionals is imperative to ensuring these entitlements are upheld. There are role plays and small group sessions where participants grapple with real-life cases to tease out the complexities involved in human rights violations in health. As the week progresses, participants divide into small working groups (replicating the process of curriculum reform teams) to develop specific educational competencies and materials that trainees could then implement at their respective institutions. As well, the challenges of institutional transformation in a fledgling democracy after years of racial and other forms of discrimination are discussed frankly with a variety of representatives from government, para-statal institutions whose mandate it is to protect human rights, civil society organisations, senior academics and struggle activists in order to give participants a sense of stakeholder investments in curriculum reform. In later years, the course has involved past participants of previous TTT courses who present their own experiences with implementing educational activities in health and human rights and provide recommendations for current trainees, with a long-term aim of facilitating the formation of a network of alumni. In addition to intense classroom instruction and dialogue, participants keep a reflective journal of their experiences and perceptions throughout the week, monitor the media for current events in health and human rights, and undertake field trips (e.g. to Robben Island, which is now a museum documenting the life experiences of political prisoners incarcerated there for decades, including former President Nelson Mandela).

In order to assess the outcomes and impact of this training, the authors undertook an evaluation to determine how well the course prepared participants to return to their institutions and implement educational reform. The objectives were to: describe the profile of course participants; document their experience prior and after the training course; and, identify what factors enabled or obstructed the implementation of human rights teaching at their individual institutions following the course. By following up past trainees of the course to ascertain what they were doing with regards to human rights training and what barriers or obstacles they encountered in their efforts, it is hoped that gaps would be identified to develop further training and to offer support to improve the ability of teachers to teach human rights to health professional students [18].

## Methods

The study sought to trace all 162 past participants of the eight Train the Trainers courses held from 1998 - 2006. Those who could be contacted were first notified by telephone of the purpose of the study and asked to confirm their willingness to participate. Subjects were then sent a comprehensive questionnaire (herewith called the "TTT survey"; see Additional file 2) by e-mail (preferred and predominant method), fax, or post and requested to return the completed questionnaire within six weeks. Participants were sent several reminders, and the deadline was extended on numerous occasions to accommodate participants' busy faculty schedules and holiday leave. Attempts were made to find participants who had since relocated from their prior job by questioning former supervisors, coworkers, and acquaintances from the same course year as well as by searching for updated contact details on the internet. Questionnaires were collected at UCT by a research intern (EGE) from June to September 2007. Four participants returned an earlier version of the questionnaire that they were given in 2006 when the study was first piloted, and thus data regarding their responses to certain questions may vary slightly from those of the other participants.

The survey format consisted of closed-ended questions about formal and informal curriculum implementation, reasons for attending the Train the Trainers course, and educational endeavors since attending. In addition, a section was devoted to Likert scale questions with the answers ranging from "strongly agree" to "strongly disagree" (for the purposes of analysis, "strongly agree" and "agree" were combined, and the same was done for "strongly disagree" and "disagree."). Finally, qualitative assessment was sought through open-ended questions on perceived barriers and allies in instituting health and human rights educational activities.

Ethics: Ethical approval was obtained from the University of Cape Town Faculty of Health Sciences Research Ethics Review Committee (ERC) and the Stanford University School of Medicine Institutional Review Board (IRB). Questionnaires were anonymous and participants were assured of the confidentiality of their responses.

### Data Analysis

Quantitative data were entered and analysed using Microsoft Office Excel, XP Professional version. Continuous data were summarized using means, medians, standard deviations and ranges and categorical data using frequency distributions and proportions. Comparisons of means were done using t-testing unless the data were non-normally distributed, in which case, comparisons of medians were done using the Mann-Whitney U test. Categorical data were compared using the χ ^2 ^test. For analysis of reported teaching activities, a blank answer was interpreted as a negative response (no teaching activities undertaken). Results based on persons and person-years were presented graphically through the use of Excel and with the assistance of StatistiXL for Windows Excel version 1.7. Comparison of rates was conducted based on an assumption of a Poisson distribution. A content analysis of qualitative data, which included careful reading and re-reading of written responses and narrative comments in the questionnaire, was conducted identifying common themes and designated key words. Analysis was conducted by one researcher (EE) with support from other co-investigators. This analysis allowed for identification of specific barriers to, and facilitatory factors for, curriculum reform.

## Results

Out of 162 past participants, 46 (28%) completed and returned the TTT survey. Recent alumni, (those participants who attended the Train the Trainers course in the two years preceding the data collection (2004-2007) were more likely to return the TTT survey than earlier (2004 or earlier) alumni (44% vs. 21%, respectively; p < 0.001). Thirty six past participants could not be contacted; therefore, TTT surveys were sent to only 126 past participants, for a response rate of 37% of those contactable. Loss to follow up was attributable to death (n = 3); relocation nationally (n = 3) and abroad (n = 4); disconnected phone lines and/or obsolete email addresses (n = 26). Reasons given for non-completion of the survey, despite repeated encouragement to do so, include survey length, lack of time, perceived lack of relevance to their current job (one respondent reported no longer working in an academic institution), maternity leave, other life circumstances, or some combination of the above (Table 2).

**Table 2:** Survey completion rate of past participants of the Train the Trainers course*

Year	No. Attended	Lost to follow-up	Survey Completion		Completion Rate (%)
			Completed	Did not complete	
1998	24	12	2	10	8%
2000	7	1	3	3	43%
2001	9	0	1	8	11%
2002	17	5	3	9	18%
2003	25	4	8	13	32%
2004	28	9	6	13	21%
2005	30	5	9	16	30%
2006	22	0	14	8	64%
Total (%)	162	36 (22%)	46 (28%)	80 (49%)	28%

* The TTT course was not held in 1999.

The total person-years since attending the TTT course of survey respondents was 142. The contribution of past participants to person years of follow up by different cohorts was more or less equal (between 10% and 15%) for most years in which the course was run, with the exception of respondents who did the 2003 course (23% of person years) and the 2001 course (6% of person years).

**Figure 2:**
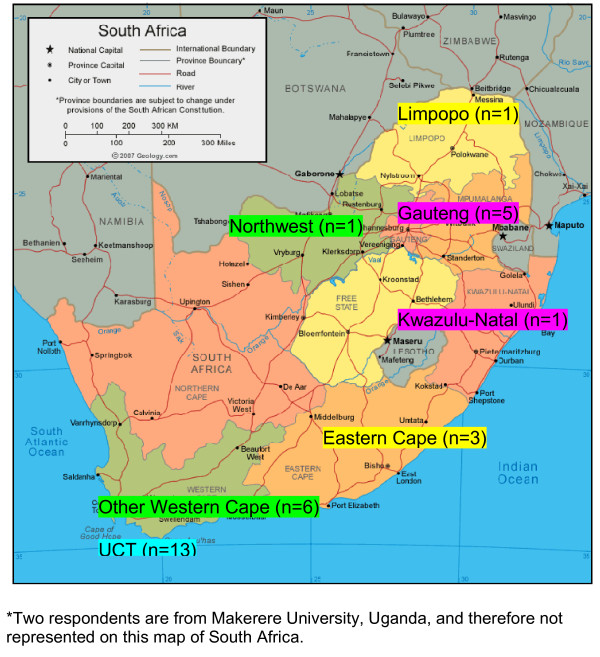
Provincial representation of academic health sciences institution affiliations of respondents*.

Thirty-one respondents (67%) reported currently working at academic institutions (medical schools, nursing colleges, etc). One respondent was retired from a university faculty position at the time of survey completion, and the remaining fourteen trainees were employed in non-academic settings in the health sector (such as hospitals, NGOs, research centers, and public health departments). Figure 2 shows the geographical distribution of respondents, based on their institutional affiliation at the time of the survey. Table 3 further describes the job responsibilities of respondents.

**Table 3:** Current Job Responsibilities of Trainee Respondents

Title	N (total = 46)
**Academic/educational (n = 31)**	
*University Lecturer, Course Facilitator, Professor*	*21*
Lecturer (Junior and Senior)	
School of Pharmacology Professor	
Vice Principal	
*Nurse Educator*	*4*
Hospital	
University	
*Student (MPhil or Medical)*	*2*
*Institutional HIV/AIDS coordinator *	*1*
*Other (Researcher, Psychologist, Not Specified)*	*3*
	
**Non-governmental (n = 4)**	
*Chairperson *	*1*
*Project Officer*	*1*
*Nongovernmental Staff *	*2*
**Research (n = 4)**	
*Researcher/Investigator*	*2*
*Research Advisor on Ethics, HR, and Law*	*1*
*Occupational Health and Social Auditor on farms/Researcher*	*1*
	
**Health service providers or managers (n = 5)**	
*Chief Dentist, Dept of Health*	*1*
*Medical Intern*	*2*
*Hospice Chief Executive Officer*	*1*
*Call Center Manager, Dept of Health*	*1*
	
**Other (n = 2)**	
*Legal Advisor, private hospital group*	*1*
*Not Specified*	*1*

Trainees reported hearing about the Train the Trainers course in many different ways. The most common source was electronic mail announcement (32%), followed by coworkers, supervisors, past participants, professional publication, local press, and other sources. The majority of respondents (65%) indicated a general interest and/or curiosity in human rights as the primary impetus for attending the TTT course. Some further noted a desire to learn about human rights issues regarding disease outbreaks, specifically with regard to refugee health, migrant labor and poor living conditions; to know about rights-based approaches to promoting health and how to implement these; to align teaching and learning to South Africa's Patients' Rights Charter and thereby make it more applicable to the community; to gain further insight into bioethics and health and human rights (HHR) education; to increase their advocacy roles with fellow staff at their home institutions; to expand a HHR network in Africa; and to recognize a personal disability and a desire to empower others. Fifteen respondents (33%) additionally noted that they were sent by their home institution in an effort to enhance human rights education. Eleven respondents mentioned they were already teaching human rights. Two of these eleven, as well as six others (n = 8 or 35%) reported that they were already teaching related material, including: ethics and/or bioethics, health law, professional practice, sexual and reproductive health, health advocacy and equity in patient care.

**Figure 3:**
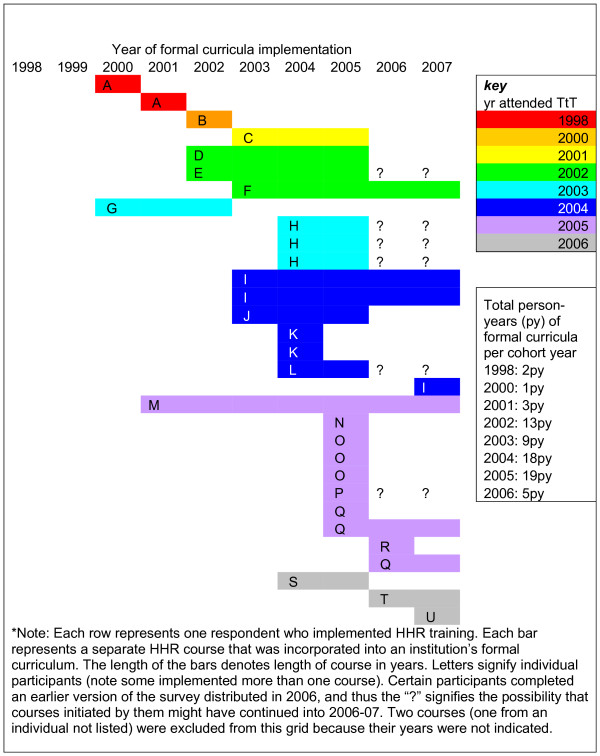
Timeline of formal curricula implementation*.

Twenty-two of the 46 respondents (48%) reported having implemented 33 *formal *health and human rights educational activities in their work environments. Of those currently working at academic institutions (defined by job description and/or university affiliation), eighteen past participants (58%) had implemented new curricula at their institutions, whereas of the 15 in non-academic settings, only four (27%) had implemented formal health and human rights curricula in their work environments (χ ^2^_1 _= 0.04, p = 0.84). Figure 3 shows the timeline of HHR course implementation. Some past participants had implemented HHR education prior to their attending the TTT course (termed "pre-course implementation" and these respondents were termed "pre-implementers"), which supports the above claim by 11 respondents that they were already teaching human rights when they attended the TTT course. Six courses were implemented by five past participants (pre-implementers) prior to attending the TTT course, with 12 course years prior to attending and 25 total course years. This may be slightly conservative given the fact that the data for four of these courses were collected from the pilot version of the TTT survey, which did not contain the years 2006 and 2007 as answer options for this question; and, the past participants who completed these questionnaires could not be contacted for clarification. The rate of pre-course implementation in the entire sample was 0.04 (12 course years/272 person years), while the rate of post-course implementation was 0.41 (58 course years/142 person years), which was statistically significant (p < 0.001, relative risk 9.26; 95% CI 5.14-16.66). For only those who were pre-implementers, there were 25 total course years for 6 courses (mean 4.2 years per course), implemented by five individuals (mean 5 course years per person). For post-implementers, there were 45 total course years for 25 courses (mean 1.8 years per course), implemented by 17 individuals (mean 2.6 course years per person). The duration of course implementation was significantly higher amongst the pre-implementers versus the post-course implementers (Mann-Whitney U test p = 0.001).

Of the 33 formal health and human rights curricular interventions, 14 were created *de novo *and 18 were adapted from existing curricula (the remaining one did not have an answer for this question). Table 4 lists the variety of ways in which human rights teaching has been incorporated into health sciences curricula by respondents as well as how learning has been achieved. Examples in the "Other" category include: morning workshops with student presentations, occasional lectures between other classes, and a theme within a module offered to third or fourth year medical students. Specific disciplines into which human rights are commonly integrated include: pediatrics, primary care, palliative care, audiology, occupational health, and public health. Other subjects taught with human rights range from sociology and clinical research to pharmacy practice and HIV/AIDS education. Target audiences include learners from various disciplines (the most common being nursing students followed by medical students, reflecting the professional mix of TTT course participants), practicing clinicians, faculty, and other staff. HHR teaching took the form of lectures, small group discussions and projects, role-plays, field trips, guest speakers, film/video, community-based learning, and experiential learning (much like the parent Train the Trainers course). Twenty-nine of the 33 programs (88%) were compulsory for the target audience; the remaining four were elective options. Assessment of learning included both formative and summative components: exams (essay, multiple choice, and oral), case studies, reflective journaling and papers.

**Table 4:** Formal integration and assessment of HHR in health sciences curricula*

	Formal Curricula
**Method of Integration**	
Taught as sole subject	3
Theme throughout curriculum	10
Module within course:	
Clinical skills	4
Communication skills	7
Bioethics	8
Professional ethos (eg: nursing)	10
Specific discipline (eg: pediatrics)	15
Other subject	15
Other	8
**Assessment of Learning**	
Essay exam	17
Multiple choice exam	10
Oral exam	2
Case studies	12
Reflective journaling	7
Papers	3
Other (eg: poster, feedback, evaluation)	13

*Note: respondents could select more than one answer.

In addition to formal curricula, respondents have also utilized extracurricular or co-curricular educational activities to implement HHR teaching at their institutions. In total, 72 extracurricular activities were implemented by 21 past participants (median number of *informal *activities = 1; range 0 to 7; Table 5). Examples of such teaching include: student leader training, discussion of HHR issues in ethics meetings, or distribution of educational pamphlets. Of note, institutional reconciliation commissions and HHR film series, two answer options in the questionnaire, were not selected by any survey respondent. Those who had not implemented formal curricula were just as likely (based on person), if not more so (based on frequency), to report adopting extracurricular teaching as those who had formally incorporated human rights. In total, 37 extracurricular activities have been started by 14 of the 24 (58%) past participants who did not implement formal HHR curricula, compared with 35 extracurricular activities by eleven of the 22 (50%) past participants who have also implemented formal HHR curricula. This difference was not statistically significant ((χ ^2^_1 _= 0.57, p = 0.45).

**Table 5:** Extracurricular (informal) educational activities in Health and Human Rights*

	Formal Curricula	
	Implemented	Not Implemented
Extracurricular Activity		
Elective experiences in HHR^+^	0	4
Special studies modules in HHR	2	0
Speaker series	2	2
Film series	0	0
Interest group	1	3
Admissions recruitment policies for disadvantaged students	3	1
Staff recruitment policies for disadvantaged groups	3	3
Research initiatives in HHR	4	2
Staff/faculty development in HHR	8	6
Emphasis on HHR-based approach in teaching	9	9
IRC° or other self-examination of role of institution under apartheid	0	0
Development of health professional oath/code of ethical conduct	1	3
Other	2	4
Total	35	37
None	7	8
No Answer	4	2

* Participants could select more than one response; ^**+**^HHR - Health and Human Rights;° IRC - Institutional Reconciliation Commission.

The persons most commonly identified as allies for human rights teaching by respondents are listed in Table 6 with the most frequent being coworkers (n = 27, or 59%), followed by departmental chairs (n = 19, or 41%), students, "other sources" (ex: director of centre, members of an institutional review board, course convener), and, finally, deans. There were no significant differences in who was considered an ally, or in the reporting of any allies versus no allies between those who implemented formal curricula compared to those who did not. However, 55% of implementers reported their Departmental Chair as an ally compared to 29% of those who were non-implementers (χ ^2^_1 _= 0.08, p > 0.9) and implementers were more likely to report each category of institutional persons (Deans, Departmental Chairs, Coworkers and Students) as potential allies than non-implementers. Overall, 47 forms of ally support were endorsed by those who reported implementing formal curricula (mean 2.47, median 2) compared to 34 without formal curricula (mean 1.89, median 1), but this difference was not statistically significant (Mann-Whitney testing; p = 0.105.) Eleven respondents (four of those with formal curricula and seven without formal curricula) did not report any form of ally support.

**Table 6:** Institutional Support and the Implementation of HHR into Formal Curricula

	Formal Curricula			
	All respondents	Implemented	Not Implemented	χ ^2#^
		(n = 22)	(n = 24)	
Institutional Allies*				
Deans	10	6	4	0.39
Departmental Chair	19	12	7	0.08
Coworkers	27	15	12	0.24
Students	14	9	5	0.14
Other	13	5	8	0.43
"None"	2	1	1	0.95
No Answer	8	3	5	0.53
Any allies	36	18	18	0.57
Institutional Support: General*				
Increased Budget	1	1	0	0.29
Provided Additional Resources	5	3	2	0.56
Formed New Committees	5	3	2	0.56
Fellowship/Internship	2	1	1	0.95
Funded Training for Staff/Students	9	4	5	0.82
Support for HHR Education Training	6	5	1	0.06
Given Sabbatical or Research Leave	1	1	0	0.29
Funded HHR Research	4	3	1	0.25
Other	7	5	2	0.17
"None"	7	5	2	0.17
No Answer	17	5	12	0.06
Any support	22	12	10	0.38

*Note: participants could select more than one response; ^**#**^comparing those who reported implementation to those who reported no implementation.

Regarding general sources of institutional support reported as valuable by respondents, training for staff and students was most commonly cited (n = 9), followed by support for additional HHR educational training (n = 4), new committees, additional resources, funding for HHR research, and a fellowship or internship in HHR. Implementers of formal curricula were slightly more likely to report some form of general institutional support (55%) than non-implementers (42%) (χ ^2^_1 _= 0.18; p = 0.67) and to report more types of general institutional support (median 1.1; range 0-6; median 0.6; range 0-3, respectively; Mann Whitney p = 0.215) but these differences were not statistically significant (refer to Table 6).

There was no relationship between the proportion of respondents reporting some form of support from allies amongst those sent by their organizations or institutions to the course (80%) compared to those who did not report being sent by their institutions (77%) (see Table 7). Seven respondents mentioned other sources of support, such as evaluation of training, renewal of contracts and funding for poster presentations. An increase in budget was a rare occurrence (one response), as was research leave or sabbatical. Some success stories were reported and were largely credited to "buy-in" from key faculty leadership. As one past participant wrote, "both the head of department and Dean were very supportive."

**Table 7:** Relationship between perceived institutional support and institutional reason for attending TTT course

	Sent by organisation*	Not sent by organisation
	(n = 15)	(n = 31)
**Some form of institutional allies**		
Yes	12	24
"None"	0	2
No answer	3	5
**Specific examples of institutional support**		
Funding for training for staff and students	4	4
Support for additional training in HHR education	0	5
Both	1	0
Neither	5	12
No answer	5	10

*Respondents self-reported being sent by his/her institution to attend the Train the Trainers course vs. those who did not report this as a reason to attend.

After taking the course, respondents reported both heightened awareness of human rights issues in healthcare and more job satisfaction (11/34 or 32% of responses). One respondent wrote that the TTT course was "empowering and enlightening." Many cited the impact of the course as more personal than career-shifting: "[t]he main effect has been a personal one for me and the way I now evaluate situations and awareness of other people." Eleven other respondents reported no particular impact on their careers but many noted they became better able to integrate HHR in the teaching they were already doing. Two participants did, however, report a change in careers as a result of the TTT course: one moved into social auditing and the health and safety aspects of farm workers, and another began working at a research training unit, stating that the TTT course, "really changed my career path and integrated aspects into my career which have made it far more rewarding to me." Another had been appointed to work on an ethics committee at her institution as a human rights advocate. One past participant wrote that the course "gave me a better understanding of what [South Africa] had overcome as a country and a people"; and, while a substantial portion of the information presented was "shocking in its reality," it provided "a sense of awe in terms of how forgiving people can be." This same participant also noted that the course "had both an emotional and psychological impact on how I relate to the people I present to and work with." One trainee summed up her experiences with the course in the following way:

"Success is more than moderate in formal setting[s] at [the] university, [while the] informal setting [has not been] very successful but very fulfilling. Students consider health and human rights as part of [the] apartheid past - [however,] when incorporated into curricula with marks attached or [illustrated with] case studies of current issues [it] garnered student interest... The Train the Trainers course gave me case histories and confidence."

There were, however, several reported negative experiences as a result of attempting to implement HHR educational reform. One participant wrote that her career had not advanced because: few faculty members have sensitivity in HHR; there are difficulties in teaching human rights outside the classroom; and, there is a lack of commitment at senior leadership level to curriculum change. Another individual, who was actually teaching material related to HHR prior to attending the course, reported that after the TTT, she became  "dismayed by the corruption in management" and, as a result, left her job since attending the TTT course. Another, who had not been teaching HHR prior to the TTT course, reported that he became more vocal about human rights violations, and consequently promotions were denied and his position of employment became jeopardized.

Overall, with regard to career development, of the respondents who were teaching either HHR (pre-implementers) or related material prior to the TTT course: eight stated a positive impact from the TTT course (such as more opportunities to teach others, more inspired to implement changes, more career advancement), eight stated no particular impact (ex: "already teaching HHR"), one had since left her job ("dismayed by corruption in management") and one did not answer the question. In comparison, of the remaining respondents: eight reported a positive impact similar to those above, seven stated no impact (ex: "unable to implement at [particular institution]"), one implied a negative impact ("more vocal thus job in jeopardy and promotions denied"), and 12 did not answer the question. Furthermore, those teaching HHR and related material equally reported a specifically positive impact on teaching HHR at their institution when compared to the remaining respondents (12 vs. 12).

**Figure 4:**
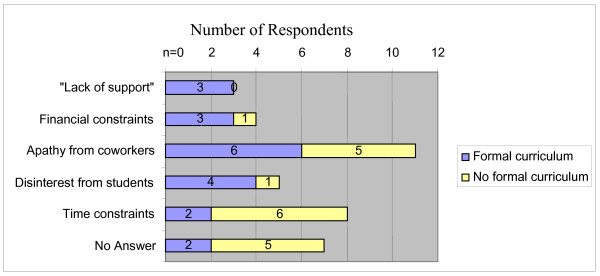
**Institutional barriers to HHR implementation**.

In regards to specific barriers in implementing HHR education, three out of 39 respondents indicated a lack of institutional support (8%). In addition, participants noted budget constraints (10%), disinterest by students (12.8%), apathy on the part of their colleagues (28%), and difficulty in finding time to implement - either in the preexisting curriculum or their own schedules (20.5%) (Figure 4). Those not formally implementing HHR education most commonly cited time constraints and apathy from coworkers as barriers. Additionally, one past participant noted the obstacles of "personal vendettas, nepotism, and overbearing bureaucracy." A former medical student, now a practicing physician, wrote that, "new ideas [regarding HHR] are viewed as arrogance" in his new environment, but he is confident that "positive changes will occur eventually." Another wrote that she was challenged by a power differential described as the "student's identity" and "personal difficulties," making it hard for the educator "because it brings who you are into the equation." She stated that the TTT course "assisted in normalizing conversations that needed to occur for a human rights approach to be taken."

**Figure 5:**
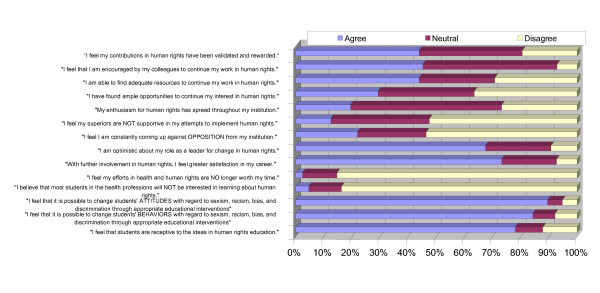
**Perceptions of past participants on support and obstacles, job satisfaction, and student receptiveness to HHR curricula implementation**.

Notwithstanding some negative experiences, participants were generally more likely to be favourable than unfavourable in assessing their ability to advance human rights education, obtain support from their institutions and secure students' interest in human rights. Figure 5 presents the attitudes past participants have in regards to their role as a leader for change in human rights, their job satisfaction as a result, and if they felt their efforts were worth their time. The majority of respondents report optimism, satisfaction, and encouragement from colleagues, as well as support from their institutions/organisations with regard to implementation of human rights.

Respondents also offered suggestions for future TTT courses, including: an increased frequency of courses per year; online teaching for those otherwise unable to attend; and increased participation by educators (including coworkers and superiors) to more effectively incorporate human rights in healthcare education.

## Discussion

### Human rights education and the Train-the-Trainers model

To date, there are many important international initiatives that establish how students from medical, nursing, and the allied health sciences must be equipped to address human rights issues, encouraged by senior professionals, and inspired to continue their education in the field [19]. As noted in Cambodia, implementing human rights reform in healthcare curricula must be both clinically relevant and pertinent to the social, political, and cultural environment in which it takes place [20]. This is equally true in South Africa, where human rights sentiment is fueled from memories of a corrupt, discriminatory, and a painfully recent apartheid past. This study provides some indication of this Train the Trainers course as an effective means of "growing" human rights education. After taking the course, there was a nine-fold increase in implementation of human rights teaching amongst survey respondents. To ensure sustainability, local staff must assume a leadership role to guarantee that any changes which are implemented are truly long-lasting [20]. Institutions which are perceived to support human rights education must follow through by prioritizing, and funding, additional staff to attend training modules to ensure sufficient coworkers are primed as allies for trainees' future curricular implementation and do not act as obstacles to change. Nevertheless, it is likely that there will continue to be institutional challenges of finding appropriately trained staff, securing sufficient funding, and overcoming the apartheid-era resistance "from staff who believe that such teaching is unnecessary and from students who either fail to attend the course or who fail to read around the subject." [21] Even if an instructor feels that a student's beliefs and attitudes may be changed with regard to racism, sexism, and other forms of discrimination, a percentage of these respondents (13%) still perceive students' disinterest in HHR as a barrier to implementing reform. Regardless of the obstacle, even where respondents were unable to report implementing HHR education at their institution or career advancement as a result of the course, survey respondents still reported significant personal growth and reflection as a result of their participation in the course.

Human rights activism is not a passive or static affair, requiring a range of teaching methods that cannot be done solely in didactic lecture format. Many of the 22 respondents who have initiated modules, courses, or other trainings in human rights use instructional activities like case studies, role plays, and media watches. With the intention of inspiring and encouraging other educators, one past Train the Trainers' participant has published data on her successes and challenges in implementing human rights into the nursing curriculum at her institution [22]. The compulsory nature, modes of evaluation, and overall structure of the 33 implemented *formal *human rights educational activities reported by respondents are derived from models taught in the parent TTT course, and thus further justify the recruitment of additional health professions faculty to attend such education modules [16,22].

### Role of Allies

Overall, about half of responding participants of past TTT courses were likely to implement human rights teaching into their institutions' formal curricula. This is encouraging given the wide range of job titles and institutional affiliations of these trainees. Those from academic institutions were able to implement formal curricula more easily than those outside of the university setting. However, those who did not formally implement health and human rights teaching are nevertheless incorporating HHR concepts informally, primarily through a holistic emphasis on HHR approaches in teaching and general staff and faculty development. Coworkers are the most commonly cited allies both for those implementing formal curricula and those who have not. Heads of department are also perceived as crucial allies for implementation, perhaps because they are in positions that can effect or support change from a higher echelon of the institution. Conversely, the data suggest that coworkers who are hostile to HHR act as barriers to curricular reform; and although not strongly evident in the data from this study, the same probably would apply in practice for departmental chairs and deans.

There was a non-significant association between the reporting of implementation of formal curricula following the course and the presence of allies and support. Failure to achieve statistical significance may be related to small sample size, since it is reasonable to anticipate that the presence of both institutional allies and general support will assist effective curriculum change. On the other hand, respondents who reported not implementing curricula were more likely to report time constraints, whether personal or within an academic course schedule, as a barrier to reform. Of course, time constraints may be a symptom of a larger institutional problem reflecting lack of support from higher levels, or may have been a convenient excuse for a range of other obstacles.

Based on the timeline (Figure 3), while most formal curricular interventions occurred within a year of the initiator attending the TTT course, it is evident that some interventions in HHR were attempted prior to attending this course. These educators likely had a prior interest in HHR, and perhaps attended the TTT course to become better equipped to implement further change. In fact, this may be a confounding factor in interpreting the impact of the course in that selection bias may be operating to attract participants more likely to pursue human rights teaching *ab initio*. Nonetheless, educators with previous HHR experience TTT may have brought with them personal successes and failures, which might have helped guide the future efforts of other participants. Further, the overall majority of interventions occurred after attending the TTT course; and, it is important to note that some were implemented several years later. This suggests that time is required to garner support - from allies such as coworkers, departmental chairs, students, and deans, and in the form of finances, time, and other support - in order to establish curriculum change, but also suggests that interventions could be conceived as sustainable if persisting long after the course.

### Barriers to Change

Those citing time constraints as a barrier to formal human rights implementation may face logistical challenges to allocating ample time for HHR education in a curriculum where medical humanities may be de-emphasized and basic sciences may take the forefront. What may be viewed as competition for curriculum time may instead be developed by a judicious collaboration with pre-existing didactic lectures and clinical training [23]. Additionally, implementation in the formal curricula may be either reinforced or undermined by the institution's hidden curriculum through prioritization, resource allocation, and even nomenclature [24,25]. Courses on pathophysiology, for example, may garner more class-time than sessions on health and human rights. More resources may be devoted to cadaver labs, clinical skills evaluations, and nationally standardized assessments and examinations. Furthermore, even if a title reflecting human rights education is in the syllabus, true change may be slow to occur if the mechanisms for support are not adequately in place.

The responding trainees who have struggled with implementing health and human rights educational activities cite many institutional barriers, such as the lack of support (financial, emotional, and otherwise) from colleagues and superiors, student apathy to discussing "things of the past," and limited time to enact meaningful initiatives. Nevertheless, many who have reported barriers still manage to incorporate HHR into both formal and informal curricula. There is a spoken need for the "power to influence change," and the necessity of training deans, departmental heads, managers, administrators, CEOs, and politicians on the core tenets of human rights in health to ensure that change also occurs from the top down while being implemented from below. Indeed, many written suggestions for future Train the Trainers courses recommend including more academic colleagues in the training to make human rights a universally recognized and necessary addition to a health sciences curriculum. True or not, there is a perception that colleagues and students believe that the past is irrelevant, or perhaps too painful, to discuss at present [10]. Just as student interest in human rights (or perceptions thereof) influence the incorporation of these themes in health professions curricula, the commitment of the faculty member and the significance they place on human rights education also undoubtedly influences whether or not this curricula is in fact incorporated and valued. Economic fragility on a national level translates to budget cuts and financial constraints institutionally, which makes any ideal human rights reform hard to implement [10,26]. Embedded in financial limitations is inadequate time to implement changes to a curriculum given other obligations, which is further propelled by lack of compensation for overtime hours.

### Limitations

The findings of this study may be subject to a number of limitations. First, the population included past participants in a course with a very broad range of follow-up time (between one and nine years). Those who more recently attended the course were more readily contactable and in general more participatory in the study. Further, the nature of the study favored participants who had frequent access to and comfort with the internet, as reminders were more quickly transmitted via e-mail than by telephone; and, responses were more reliably received electronically than through fax transmissions or postal packages. Moreover, we achieved a modest 28% overall response rate (or 37% of those who were contactable) which limits the generalisability of our study findings. The respondents may therefore represent a selected sample of the highly motivated few which would overestimate the implementation results. They are also geographically concentrated in the Western Cape province, near where the course is held annually. On the other hand, many past participants may in fact be initiating health and human rights education at their respective institutions but may be too overworked and overwhelmed to allocate adequate time to answer questions about it. Nonetheless, even if all non-responders have failed to implement any post-course human rights training (a severely conservative assumption), there is still evidence of 100 formal and non-formal training initiatives generated by the course. This remains a relatively satisfying impact reported and probably underestimates the true impact considerably. Moreover, there was no obvious imbalance of follow up time (person years) by year of taking the course, suggesting that any possible bias from specific cohort year was limited in the study.

Furthermore, many past participants working in rural settings were unintentionally excluded as a result of unsuccessful communication attempts. This "urban bias" therefore limits any unmodified application of this study's conclusions and suggests inequity with regard to technology access and distribution of information for this population. Others who voluntarily excluded themselves had changed jobs and felt they were no longer in a position to implement health and human rights teaching in their new environment. Many of those lost to follow-up had moved jobs or changed their e-mail addresses or phone numbers (without notifying their former employers). A portion of these (at least four from this study) may be part of the "brain drain" of healthcare professionals leaving sub-Saharan Africa to work in Northern countries [27]. Despite an attempt to minimize subject exclusion by updating contact information in a database at a 2006 conference at UCT - attended by many prior trainees - some former participants' phone numbers were defunct and e-mail addresses undeliverable [28].

The nature of a cross-sectional design with retrospective data collection limits the extent to which associations identified may reflect a causal relationship. However, it is unlikely that reverse causation would explain reporting of higher rates of institutional support by those implementing curriculum change, since it would be unusual for a curriculum intervention to generate institutional support unless it had been running for a good number of years already. In the cases of the few individuals who had attempted, and succeeded, in human rights curricular interventions prior to attending the TTT course, changes may have already been brewing at their respective institutions as a result of their initial pioneering efforts.

Lastly, we cannot firmly attribute the changes reported in activities, confidence and attitudes amongst respondents to the impact of the course without use of an experimental design. Participants may be pre-selected individuals with a belief in human rights education who be more likely to implement human rights education irrespective of their participation in the training course. Given the difficulties of setting up and implementing a randomized design, further research should at least compare the TTT alumni cohort with a control population of health sciences faculty who have not been through such training. Longitudinal analysis with medical, nursing, dental, and other allied health graduates who finished their training within the last ten years may assist in identifying how implementation of lessons from the TTT course are trickling down to the next generation of healthcare professionals. It would also be valuable to compare South Africa's training of trainers with what is occurring in other countries without such a tumultuous history of human rights abuses to determine if it is more or less successful in a volatile setting.

## Conclusions

Despite the official demise of apartheid as well as increased awareness and sensitivity about achieving equity, non-discrimination and fairness, violations of human rights persist in the South African health sector [3,13,29,30]. Amnesty International has outlined how excessive workload and lack of resources (financial and otherwise) compound governmental pressure on medical personnel to remain tightlipped about any violations they do see [4]. Fortunately, organisations such as the Health Professions Council of South Africa, the body responsible for setting educational norms and standards in South Africa, has mandated core competencies in medical ethics, health law, and human rights as requisite for graduation from all health sciences programmes around the country, raising the profile of human rights as an integral part of healthcare worker training [28,31]. As previously noted, it is imperative that information and experiences be shared with others in the health and human rights educational community, from local workshops and national journals to international caucuses and global inititatives [19,28]. Ultimately, South Africa's apartheid past informs, and indeed exemplifies, the need for future health professionals to engage in explicit human rights education and training.

## List of abbreviations

HHR: health and human rights; TRC: Truth and Reconciliation Commission of South Africa; TTT course: Train-the-Trainers course on Health and Human Rights for Health Professional Educators offered by UCT; TTT survey: research questionnaire used for follow-up analysis of past participants of the TTT course; UCT: University of Cape Town.

## Competing interests

LBR and LL have designed and co-convened the TTT course since its inception.

## Authors' contributions

LL and LBR conceived the original study question; all three authors contributed to the development of the study protocol, and the study instrument; LBR and EE took responsibility for identification of the study sample and distribution of the questionnaire; EE oversaw data capture; all three authors were involved in data analysis, interpretation and write up of results, including various drafts of the manuscript. All authors signed off on the final manuscript.

## Author's information

Elena G. Ewert, MD (formerly Elena Garcia) graduated from the Stanford University School of Medicine in Stanford, CA and is a past intern of Mount Sinai School of Medicine's International Exchange Program for Minority Students in New York, NY. She is currently undergoing her Residency training in Emergency Medicine at Denver Health Medical Center in Denver, CO. Laurel Baldwin-Ragaven MDCM, CCFP, FCFP is the Medical and Executive Director of the Malta House of Care, a free mobile medical clinic, is on staff at the Asylum Hill/University of Connecticut Family Practice Residency Training Program and is a Visiting Professor at the School of Public Health and Family Medicine at UCT. Leslie London MB ChB, BSc Hons (epid), DOH, MD, M.Med. (Comm Health) is Professor and Director of the School of Public Health & Family Medicine at the University of Cape Town in South Africa and Head of the School's Health and Human Rights Division.

## Pre-publication history

The pre-publication history for this paper can be accessed here:

http://www.biomedcentral.com/1472-6920/11/47/prepub

## Supplementary Material

Additional file 1 **This file provides a brief explanation of the use of race terminology in this paper**. Click here for file

Additional file 2 **Questionnaire used for data collection in this study**. Click here for file
