# Supplementary material for: Training Trainers in health and human rights: Implementing curriculum change in South African health sciences institutions
Source: BMC Med Educ. 2011 Jul 25;11:47. doi: 10.1186/1472-6920-11-47 (PMC3157433; doi:10.1186/1472-6920-11-47)
Supplement: Additional file 2 — Questionnaire used for data collection in this study. [file 1472-6920-11-47-S2.RTF]

Train the Trainers Survey

(Instructions: Please note that the grey boxes will expand to contain your answers.  Therefore, feel free to respond at length.)

Name___________________________________________________________________
Current Position/Title______________________________________________________
Institution_______________________________________________________________
Address ________________________________________________________________
Telephone __________________________        Fax _____________________________
E-mail__________________________________________________________________
Year of participation in Train the Trainers _____________________________________

Please check the accuracy of the prior information and make any corrections or additions:	     
	

1. Where did you find out about the “Teaching Human Rights for Health Professionals: Training of Trainers” course? (Tick all that apply.) 
		
 01   Past participant
 02   Electronic announcement (e.g. listserve, email):      
 03   Professional publication (please provide name):      
 04   Local press
    Workplace (please indicate):
 05   Co-worker
 06   Supervisor 
 07   Other (please specify):      


Implementing Human Rights Education and Training
	

Introduction:  The following questions address teaching and learning in Health and Human Rights.  We are interested in hearing about the creation of new courses and modules in Health and Human Rights as well as the modification of existing ones.  

Formal Curriculum	
2. Since attending the Train the Trainers course, have you started or tried to start teaching a formal educational activity (e.g. course, module, seminar, colloquium) in Health and Human Rights?

 01   Yes			 02   No     (If NO, skip to question 3, page 8)	
If yes, please complete the following table: (There is room for three activities. Each activity is one column. Questions 2.1 to 2.12 apply to a single activity. If you have been involved in more than three activities, please attach supplemental sheets, as needed.)

Name of activity:
     	Name of activity:
     
	Name of activity:
     	

2.1. For this activity, did you:


	
 01 Create something completely new 

 02 Adapt or modify an existing one (indicate that from which it was adapted.):
               	
 01 Create something completely new 

 02 Adapt or modify an existing one (indicate that from which it was adapted.):
               	
 01 Create something completely new 

 02 Adapt or modify an existing one (indicate that from which it was adapted.):
               	

2.2. In what years have you run this activity? (Tick all that apply.)
	
 01 1998               05 2002
 02 1999               06 2003
 03 2000               07 2004
 04 2001               08 2005
	
 01 1998               05 2002
 02 1999               06 2003
 03 2000               07 2004
 04 2001               08 2005
	
 01 1998               05 2002
 02 1999               06 2003
 03 2000               07 2004
 04 2001               08 2005
	

2.3. How is Health and Human Rights content taught in this activity? (Tick one.)


	
 01  As the sole subject (ie. stand-alone subject).  (If YES, skip to question 2.5) 

 02  As a module within a course containing other subjects
 
 03  As a theme integrated throughout the entire curriculum 

 04  Other (please specify):  
                  
            	
 01  As the sole subject (ie. stand-alone subject).  (If YES, skip to question 2.5) 

 02  As a module within a course containing other subjects
 
 03  As a theme integrated throughout the entire curriculum 

 04  Other (please specify):  
                  
            	
 01  As the sole subject (ie. stand-alone subject).  (If YES, skip to question 2.5) 

 02  As a module within a course containing other subjects
 
 03  As a theme integrated throughout the entire curriculum 

 04  Other (please specify):  
                  
            	

2.4. If Health and Human Rights is taught with other subjects or disciplines, indicate which ones. (Tick all that apply.)
	
 01  Bioethics

 02  Clinical skills

 03  Professional ethos

 04  Communication skills

 05  Specific disciplines, such as: surgery, primary health care, obstetrics, etc.  (Indicate which ones.):       

 06  Other (please specify):
                  
	
 01  Bioethics

 02  Clinical skills

 03  Professional ethos

 04  Communication skills

 05  Specific disciplines, such as: surgery, primary health care, obstetrics, etc.  (Indicate which ones.):       

 06  Other (please specify):
                  
	
 01  Bioethics

 02  Clinical skills

 03  Professional ethos

 04  Communication skills

 05  Specific disciplines, such as: surgery, primary health care, obstetrics, etc.  (Indicate which ones.):       

 06  Other (please specify):
                  
	

2.5. Who is the target audience for this activity? (Tick all that apply.)


( continued)

Who is the target audience for this activity?  (Tick all that apply.)
	
 Students:
      11   Medical
      12   Nursing
      13   Dental
      14   Physiotherapy
      15   Multi-professional
      16   Other (please specify):      
                        

 02   Practising clinicians 
 03   Faculty
 04   Other staff development (please specify):      

 05   Other target audience (please                        specify):       
	
 Students:
      11   Medical
      12   Nursing
      13   Dental
      14   Physiotherapy
      15   Multi-professional
      16   Other (please specify):      
                        

 02   Practising clinicians 
 03   Faculty
 04   Other staff development (please specify):      

 05   Other target audience (please                        specify):       
	
 Students:
      11   Medical
      12   Nursing
      13   Dental
      14   Physiotherapy
      15   Multi-professional
      16   Other (please specify):      
                        

 02   Practising clinicians 
 03   Faculty
 04   Other staff development (please specify):      

 05   Other target audience (please                        specify):       
	

2.6. At what point in training is this activity taught?

	
 01   Early (first third of training)

 02   Middle (second third of training)

 03   Late (last third of training)

 04   Continuing Professional Development (CPD)
	
 01   Early (first third of training)

 02   Middle (second third of training)

 03   Late (last third of training)

 04   Continuing Professional Development (CPD)
	
 01   Early (first third of training)

 02   Middle (second third of training)

 03   Late (last third of training)

 04   Continuing Professional Development (CPD)
	

2.7. Is this activity: (Tick one)


	
 01   Compulsory (e.g. a core requirement)

 02   Optional 
	
 01   Compulsory (e.g. a core requirement)

 02   Optional 
	
 01   Compulsory (e.g. a core requirement)

 02   Optional 
	

2.8. Has this activity received South African Qualifications Authority (SAQA) approval?
	
 01   Yes

 02   No
	
 01   Yes

 02   No
	
 01   Yes

 02   No
	

2.9. What instructional strategies are used in this activity?  (Tick all that apply.)


(continued) 

What instructional strategies are used in this activity?  (Tick all that apply.)
	
 01   Lectures

 02   Small group discussions

 03   Small group projects

 04   Field trips

 05   Film/video

 06   Guest speakers

 07   Community based learning

 08   Experiential learning (please specify):      

 09   Other (please specify):      	
 01   Lectures

 02   Small group discussions

 03   Small group projects

 04   Field trips

 05   Film/video

 06   Guest speakers

 07   Community based learning

 08   Experiential learning (please specify):      

 09   Other (please specify):      	
 01   Lectures

 02   Small group discussions

 03   Small group projects

 04   Field trips

 05   Film/video

 06   Guest speakers

 07   Community based learning

 08   Experiential learning (please specify):      

 09   Other (please specify):      	

2.10. What forms of assessment are used to evaluate learning in this activity? (Tick all that apply.)


 	
 01   Essay exam

 02   Multiple choice exam

 03   Oral exam

 04   Case studies

 05   Papers

 06   Reflective journaling

 07   Other (please specify):      	
 01   Essay exam

 02   Multiple choice exam

 03   Oral exam

 04   Case studies

 05   Papers

 06   Reflective journaling

 07   Other (please specify):      	
 01   Essay exam

 02   Multiple choice exam

 03   Oral exam

 04   Case studies

 05   Papers

 06   Reflective journaling

 07   Other (please specify):      	

2.11. Is there an evaluation of this activity in place?  If yes, please attach a copy of the tool.	
 01   Yes

 02   No
	
 01   Yes

 02   No
	
 01   Yes

 02   No
	

2.12. Are there established learning objectives and/or core competencies for this activity?  If yes, please attach a copy of the course outline or other material. 
	
 01   Yes

 02   No
	
 01   Yes

 02   No
	
 01   Yes

 02   No
	


Extra-Curricular Activities 
The following questions address elective experiences, special studies modules and other extra-curricular activities in Health and Human Rights.  We would like to know about any initiatives you have begun that incorporate Health and Human Rights outside the formal curriculum.   

3.0. Since your completion of the Train the Trainers course, have you incorporated or tried to incorporate Health and Human Rights in your institution in ways other than a formal course?

 01   Yes			 02   No     (If NO, skip to question 4)	

3.1. Below are some of the ways that educators have used to address human rights issues in health outside of a formal course.  Tick all that have been used in your setting. 

 01   Elective experiences in human rights
 02   Special studies modules in human rights
 03   Human rights speaker series
 04   Human rights film series
 05   Health and human rights interest group
 06   Admissions policies to recruit educationally disadvantaged students 
 07   Staff recruitment policies to achieve equity targets in historically disadvantaged groups  (e.g. race, gender, sexual orientation, disability)

 08   Research initiatives in health and human rights
 09   Methods of teaching and learning that emphasise a human rights based approach (e.g. using a patient centred approach to clinical teaching; emphasising the socioeconomic context of health and illness)

 10   Staff/faculty development in human rights	
 11   Institutional Reconciliation Commission or other self-study initiative that examines the role of the institution under apartheid

 12   Development of a new health professional oath or code of ethical conduct

 13   Other (please specify):      

Institutional Support for Health and Human Rights Education and Training
The following questions address the institutional culture surrounding your initiatives in Health and Human Rights education and training.  We would like to know about your institution's response(s) to your (proposed) projects in Health and Human Rights.

4. Who have been your allies in implementing Health and Human Rights education in the institution? (Tick all that apply.)

 01   Dean
 02   Departmental chair or head
 03   Co-workers (faculty/staff)
 04   Students
 05   Other (please specify):       


5. How has your institution shown its support of your initiatives in Health and Human Rights? (Tick all that apply.)

 01   Increased the budget
 02   Provided you with additional resources (e.g. staff, office space, computers)

 03   Formed new committees
 04   Established a fellowship or internship
 05   Funded training for staff or students
 06   Provided support for additional training in Health and Human Rights education

 07   Given a sabbatical or study or research leave
 08   Funded Human Rights research 
 09   Other (please specify):      


6. Please respond to the following statements by ticking the box that indicates your most appropriate response: 
 
 Since attending the Train the Trainers course:	Agree                                                         Disagree
strongly     Agree      Neutral     Disagree   strongly	
6.1. I am more aware of Health and Human Rights.	    01       02      03       04      05	
6.2. I feel my contributions in Human Rights have been validated and rewarded.	    01       02      03       04      05	
6.3. I feel that my superiors are not supportive in my attempts to implement Human Rights.  	    01       02      03       04      05	
6.4. I am optimistic about my role as a leader for change in Human Rights.	    01       02      03       04      05	
6.5. I have found there are too few health professionals interested in Health and Human Rights advocacy in South Africa. 	    01       02      03       04      05	
6.6. I feel that I am encouraged by my colleagues to continue my work in Human Rights. 	    01       02      03       04      05	
6.7. I am able to find adequate resources to continue my work in Human Rights.	    01       02      03       04      05	
6.8. I feel that I am constantly coming up against opposition from my institution.	    01       02      03       04      05	
6.9. I feel that students are receptive to the ideas in Human Rights education. 	    01       02      03       04      05	
6.10 I feel that bioethics has not adequately addressed human rights concerns in South Africa.	    01       02      03       04      05	
6.11. I feel that my better understanding of Human Rights has enhanced my teaching skills and knowledge.	    01       02      03       04      05	
6.12. With further involvement in Human Rights, I feel greater satisfaction in my career. 	    01       02      03       04      05	
6.13. I have found ample opportunities to continue my interest in Human Rights. 	    01       02      03       04      05	
6.14. I feel my efforts in Health and Human Rights are no longer worth my time. 	    01       02      03       04      05	
6.15. My enthusiasm for Human Rights has spread throughout my institution. 	    01       02      03       04      05	
6.16. I feel that to teach human rights to health professionals in South Africa, it is important to understand the past history of discrimination under apartheid.	    01       02      03       04      05	
6.17. I believe that most students in the health professions will not be interested in learning about human rights.	    01       02      03       04      05	
6.18. I believe that human rights and bioethics are essentially the same concepts when applied to health professionals.	    01       02      03       04      05	
6.19. I feel that it is possible to change students' attitudes with regard to sexism, racism, bias and discrimination through appropriate educational interventions. 	    01       02      03       04      05	
6.20. I feel that it is possible to change students' behaviours with regard to sexism, racism, bias and discrimination through appropriate educational interventions. 	    01       02      03       04      05	


Reflections on the Train the Trainers Course
	


7. What were your reasons for attending the Train the Trainers course? (Tick all that apply.)

 01   General interest in Human Rights
 02   Already teaching Human Rights
 03   Already teaching related material (please specify):      
 04   Researcher in Human Rights related field
 05   Concerned about research ethics
 06   Sent by organisation
 07   Curiosity
 08   Heard about it from a previous participant and felt it would be worthwhile
 09   Other (please specify):      

8.  Please rank the following five teaching activities used in the Train the Trainers course in terms of their usefulness in helping you implement Health and Human Rights into your courses. 
	Order:
1= least useful
5 = most useful

     	Case vignettes (e.g. role play, case studies)	
     	Health professional institutions' presentations (e.g. HPCSA, SANC, SAMA)	
     	TRC Submissions	
     	Curriculum development inputs and activities (e.g. assessment; framing educational outcomes, etc.)	
     	Resources presented for teaching (books, pamphlets, articles, references, videos, websites provided on the course)	


9. Have you attended any other courses or workshops on Health and Human Rights besides the Train the Trainers?

 01   Yes			 02   No     	

9.1. If yes, please include the name of course, facilitators, dates, and any other helpful information (such as the strengths of the programme/course):
     


10. What have been your best tools for continued information concerning Health and Human Rights?  (Tick all that apply.)

 01   Journal (please specify):      
 02   Internet site (please specify):      
 03   List server (please specify):      
 04   Conference (please specify):      
 05   Seminars/workshops (please specify):      
 06   Other (please specify):      

11. Would you find a booster session helpful?
 01   Yes			 02   No     	

11.1. What needs would you like to be addressed?
     


Personal Impact

11. Please take some time to reflect upon your experiences of attempting to teach Health and Human Rights in both formal and informal settings.  How would you rate your success?  What kinds of obstacles or barriers did/do you face?  How did/do you deal with them?  How well did the Train the Trainers course equip you to deal with these obstacles?  How can we improve the Train the Trainers course to better address these barriers?  What are current needs for implementing Health and Human Rights education?  (The grey box will expand to contain your answer, therefore please feel free to fill the page.)
   
     

12.  Please take some time to reflect upon your career trajectory since completion of the Train the Trainers course.  How has your personal career been affected since attending the Train the Trainers course?  How do you think your involvement in Health and Human Rights education and training affected your career?  Please indicate if you have changed post, job or direction, and if you have experienced any promotion, and the year in which these changes took place.  (The grey box will expand to contain your answer, therefore please feel free to fill the page.)

     
